# Social Environmental Predictors of COVID-19 Vaccine Hesitancy in India: A Population-Based Survey

**DOI:** 10.3390/vaccines10101749

**Published:** 2022-10-19

**Authors:** Srikanth Umakanthan, Maryann M. Bukelo, Mario J. Bukelo, Sonal Patil, Naveen Subramaniam, Ria Sharma

**Affiliations:** 1 Department of Para-Clinical Sciences, Faculty of Medical Sciences, The University of the West Indies, St Augustine 685509, Trinidad and Tobago; 2 Department of Anatomical Pathology, Eric Williams Medical Sciences Complex, Mount Hope 100607, Trinidad and Tobago; 3 Department of Paediatrics, Father Muller Medical College, Mangalore 575002, India; 4 Department of Community Medicine, RRN Hospital and Research Center, Madurai 625501, India; 5 Medical Resident, RRN Hospital and Research Center, Madurai 625501, India

**Keywords:** vaccination, intention, variables, sources

## Abstract

**Background**: During the ongoing COVID-19 pandemic, trust within a community in the projected schemes or strategies to combat COVID-19 depends on the confidence generated and launched by the government and medical employees toward the public. The “vaccination intention” within a community is determined by a range of factors, which include sociodemographic features, personal beliefs, and attitude toward vaccination. **Methods**: A nationwide survey was conducted involving 2000 people using a Tencent questionnaire platform. One-way ANOVA was conducted for age, education, and occupation with vaccination intention for the COVID-19 vaccine. Correlation analysis was conducted between sources, trust, and vaccination intention indicating both types of sources (official and unofficial sources) and both types of trust (trust in the social environment and in vaccines). **Results**: The reception of the sources of information on the COVID-19 vaccine was significantly higher from official sources (M = 5.54, SD = 1.37) and government officials (M = 5.68, SD = 1.499) compared with that from experts in medicine (M = 5.39, SD = 1.511). Among the unofficial sources, “chatting and communicating with family and friends” scored the highest (M = 4.84, SD = 1.649). In the statistics on people’s trust in all aspects involved in vaccines, the level of trust in the social environment was significantly higher and more concentrated than in vaccines (M = 5.67, SD = 1.129). The level of trust in government (M = 5.80, SD = 1.256) was slightly higher than in medical personnel (M = 5.53, SD = 1.199). People’s willingness to be vaccinated was generally high (M = 78.15, SD = 22.354). The demographic factors were not influential in vaccination intention. Both sources (official and unofficial sources) and trust (trust in the social environment and in vaccines) are significantly and positively correlated with vaccination intention. Information receptions from official and unofficial sources were significant positive predictors of trust in the social environment, but they were not significant predictors of trust in vaccines. The mediating effect of trust in vaccines on the relationship between receiving information from official and unofficial sources and vaccination intention was insignificant. **Conclusions**: This study revealed that trust in the environment is an important channel linking people’s information reception and vaccination intention, explores a new path for health information communication, and attempts to provide new ideas for health information dissemination and promotion.

## 1. Introduction

Vaccine hesitancy is defined by the World Health Organization (WHO) as “a delay in acceptance or refusal of vaccines despite the availability of vaccination services” [1,2]. Studies in India (36%), Canada (20%), and the United States (25%) have published population-based studies on COVID-19 vaccine hesitancy demonstrating that the factors that are responsible for vaccine hesitancy range from social demographics, occupation, religious beliefs, and social and environmental trust [2,3,4].

Trust is a very important factor in the study of factors influencing vaccine hesitancy. A literature review of European studies from 2006 to 2014 reveals that the main concern over the decade was vaccine safety [5]. Taylor et al. revealed that vaccine refusal was strongly associated with vaccine distrust [6]. Under the condition of the COVID-19 pandemic, vaccination intention has been shown to be positively associated with the perceived persistence of SARS-CoV-2 [7]. In addition, to determine the best way to increase influenza vaccination rates, the confidence in physicians and national health departments was assessed in 2018 [8,9]. Health-care providers are a key part in influencing public trust in scientific and epidemiological evidence [10]. Overall, regarding the trust and factors that influence vaccine hesitancy behavior among a population, the literature studies have generally focused on health professionals and trust in vaccines, such as infectious disease specialists, medical personnel, policymakers, governments, and vaccine provider systems. 

Numerous researchers have constructed models of the factors influencing the phenomenon of vaccine hesitancy, and the classification of trust has been elaborated and refined. In 2015, the WHO Strategic Advisory Group of Experts on Immunization grouped the factors into the following three aspects: (1) contextual factors, (2) individual and group influences, and (3) vaccine- or vaccination-specific issues [11]. Many Indian scholars have also analyzed and summarized the model of factors influencing vaccine hesitancy. Umakanthan et al. found that vaccine hesitancy is comprehensive and context-specific [2]. Zhang et al. suggested that vaccine hesitancy is influenced by confidence, that is, by trust in the efficacy and safety of the vaccine and in the health service system that provides it [12]. Xu et al. classified the factors influencing vaccine hesitancy into social environmental, individual or group, and vaccination service factors [13]. Yu et al. developed a framework of influences on vaccination based on the Indian population, categorizing the influences as vaccine confidence and accessibility [11] factors [13]. 

Regarding the factors influencing the phenomenon of vaccine hesitancy, trust in the vaccine itself is the focus of research, whereas research on trust in the environment other than the vaccine mainly remains at the theoretical level. In terms of trust in the environment, researchers have focused more on trust in government policies and medical personnel, whereas there are fewer studies worldwide on community and family factors mentioned by Indian researchers. Therefore, the trust in government and medical personnel, being part of the environment trust, was taken as the research object and defined as “trust in the social environment” to distinguish it from trust in the community and family. In addition, trust in the vaccine itself and trust in the social environment (including trust in the government and in medical personnel), being parts of the trust that influences vaccination intention, were included as the subjects. The following research hypotheses were formulated:

**H1a.**  *There is a significant positive association between trust in vaccines (safety and efficacy) and vaccination intention toward COVID-19 vaccines.*

**H1b.**  *In the context of the COVID-19 pandemic, there is a significant positive association between trust in the social environment (including government and medical personnel) and vaccination intention for COVID-19 vaccines.*

Since the emergence of the concept of vaccine hesitancy, the media has received research attention for its influence on vaccine hesitancy. Umakanthan’s model of vaccine hesitancy includes trust in communication and media, and the media included traditional media, the Internet, and social media and anti-vaccination activists [2]. Some findings suggest that social media may be an effective intervention tool to help parents to make informed decisions about vaccination for their children [14]. Social media applications have a positive impact on people’s vaccination behavior by being informed about vaccines [15]. The Internet is an important source for Canadian parents to find and share information about vaccines and is significantly associated with negative parental perceptions of vaccine risks [16]. Studies in India have also confirmed that information reception from the media has a significant impact on vaccination intention. Studies on the influenza A vaccine have demonstrated that news involvement has a significant positive effect on the level of understanding [17]. Studies on HPV vaccination intention suggest that media exposure does influence intention to receive the vaccine [18]. In addition, some studies have explored the impact of benefit targets in health communication strategies on influenza vaccination behavior in college student populations [19].

Currently, studies on information reception in relation to vaccine hesitancy are mainly categorized based on media channels. However, in the Internet environment, multiple sources with different identities coexist in each media channel and simultaneously disseminate the vaccine information. Therefore, the identities of information disseminators should be considered to optimize the way current researchers categorize information sources. This study classified the sources of information reception into two categories, official and unofficial sources, and proposes the following research hypotheses using authority as a classification criterion.

**H2a.**  *There is a significant positive correlation between exposure to information (official sources) and vaccination intention for COVID-19 vaccines.*

**H2b.**  *There is a significant negative correlation between exposure to information (unofficial sources) and vaccination intention for COVID-19 vaccines.*

It has been revealed that different sources of information affect people’s degree of trust. The public is more impressed with information from media sources, such as television and the Internet, and trusts information from “friends and family” sources [20] more. The largest source of information about breaking news is news websites, and trust in this source is generally higher [21]. People’s degree of trust in different sources varies. Netizens have the highest trust in national media network platforms and local government websites [22]. There is a positive correlation between the interpersonal communication or social media use and trust in rumors on the pandemic during the COVID-19 pandemic [23]. This study proposed the following research hypotheses on the relationship between information exposure and trust among various aspects:

**H3a.**  *There is a significant positive association between exposure to media information (official sources) and trust in COVID-19 vaccines.*

**H3b.**  *There is a significant positive association between exposure to media information (unofficial sources) and trust in COVID-19 vaccines.*

**H3c.**  *There is a significant positive relationship between exposure to media information (official sources) and trust in the social environment.*

**H3d.**  *There is a significant positive relationship between exposure to media information (unofficial sources) and trust in the social environment.*

Several studies have found that trust is a mediator in the relationship between exposure to media and behavioral intention. Political trust has been shown to be a mediating variable between exposure to media and subjective well-being [24]. Using a health belief model as a mediator, a study determined that both social media expression and acceptance were effective in encouraging enhanced health intention, but only by increasing self-efficacy and perceptions of severity [25]. In addition, Indian adult women’s exposure to social health information and media-based health information using WeChat increased their psychological expectation of HPV vaccination behavior, which, in turn, contributes to the intention of HPV vaccination behavior [26]. Based on the above theory and research practice, this study presented the following hypothesis:

**H4.**  *Trust among vaccines and social environment mediates between information reception and willingness to be vaccinated.*

In the research framework of this study, the independent variables are the different sources of information reception, which are classified into two categories: official sources and unofficial sources using authority as the classification criterion. The mediating variable is trust, which is divided into trust in vaccines and in the social environment. The dependent variable is vaccination intention.

Data were collected by questionnaires to first conduct a descriptive analysis of the current situation of the study population in terms of information reception about the COVID-19 vaccine from various sources and the current situation of trust from all sides and vaccination intention behaviors. On this basis, the correlations between the reception from different sources and trust or vaccination intention and the correlation between different categories of trust and vaccination intention were explored. Whether trust in the safety and efficacy of the vaccine and trust in the social environment were the mediating factors between the information reception from different sources and vaccination intention was then analyzed.

## 2. Material and Methods

### 2.1. Data Collection

This study adopted a questionnaire survey (as shown in the Supplementary File S1), which was conducted from November to December 2021. Survey questions were constructed with available questionnaire construction information and guidelines from a group of specialists and government officials, totaling ten members. The questionnaire was created and distributed in the English language. The questionnaire incorporated four significant dimensions: demography, source of information, trust, and concerns. The complete questionnaire was limited to 12 questions in total. To facilitate a detailed and better response, questions were developed in categorical (one-optional and multi-optional) and open-ended formats. The clarity of the questionnaire was tested in a pilot study among ten random individuals to confirm that the target audience understood the questions. The questions and domains were reviewed for suitability, applicability, relevance, and accuracy by experts comprising social scientists, epidemiologists, and medical doctors. The perplexing and challenging questions were then improved or omitted before the initiation of the study. Data from the pilot study were excluded from the results. A total of 2000 questionnaires were randomly distributed to people aged 18 to 60 in 20 states, autonomous regions, and municipalities directly under the central government in India using the response panel database of the Tencent questionnaire platform. The sample size was determined by the prevalence of vaccine hesitancy as determined by Umakanthan et al. [2]. To ensure the quality, questionnaires completed within 90 s were deleted, and those with missing values and with the same answers to many items were also excluded. The final valid sample size was 1748, with an effective rate of 87.4%.

### 2.2. Measurement of Variables

#### Factor Analysis of Independent Variable

The frequency of information reception about the COVID-19 vaccine from different sources is adopted as the independent variable, and the information sources of the COVID-19 vaccine were measured. There were seven question items in two major categories. Specifically, official sources include (1) information disclosure by the government and (2) public voices of experts and scholars in medical and other fields. Unofficial sources include (1) online opinion leaders (e.g., Internet-verified celebrities from the social media platform), (2) Internet users’ opinions, (3) gossip, (4) overseas institutions (e.g., foreign governments, research institutions, media), and (5) chatting with family and friends, both online (e.g., WeChat, phone call) and offline (face-to-face). All items were scored on a scale ranging from 1 (“never”) to 5 (“very often”). The average score of all items in each group was the respondent’s source receptivity score for that group. The subscales of unofficial and official sources were analyzed via factor analysis and principal component analysis (PCA), as shown in Table 1 (Cronbach’s alpha = 0.830, KMO = 0.802, Bartlett’s sphericity test *p* = 0.000 < 0.05, indicating that the factor analysis was valid, and the factors explained a total of 64.522% of the variance).

### 2.3. Dependent Variable

The dependent variable was behavioral intention, that is, people’s intention to receive the COVID-19 vaccine. After two questions to exclude those who were not suitable for the vaccine and those who were already vaccinated at the time of completing the questionnaire, it was measured by a question item, “Do you intend to receive the COVID-19 vaccine in the future?” A scoring system was used in which respondents could select any integer score between 0 and 99 out of 100, with 0 being no intention to receive the vaccine and 99 being going to receive it. A total of 12 responses were excluded due to the above-mentioned response.

### 2.4. Factor Analysis of Mediating Variable

The mediating variable is trust. In 2019, the scholar Yu Meng Ke proposed the first vaccine hesitancy model based on the Indian context, dividing confidence in vaccines into contextual factors and vaccines themselves [11]. This study referred to this model and divided the trust factors into trust in vaccines and that in the social environment. Specifically, the former was measured by the scale [27] designed in 2011, which measured trust in the safety and efficacy of vaccines. The question items were screened according to the specific situation of the COVID-19 pandemic in India, and a pretest of reliability was conducted. In addition, the scale was linguistically modified to a small extent with a high internal consistency (Cronbach’s α = 0.702). Trust in the social environment was categorized into trust in vaccines policy and that in medical personnel using the classification proposed by Umakanthan in 2021 [2]. The national policies that equate to the success of the country’s immunization program depend on disease surveillance, pathogen detection, incidence levels for mass vaccination, development and/or procurement of vaccines, choice of selective versus universal immunization, cost–benefit analyses, and resource mobilization. India is under the WHO’s Expanded Programme of Immunization (EPI) to combat six childhood vaccines, namely Bacillus Calmette–Guerin, Tetanus toxoid, Diptheria Pertussis tetanus, Diptheria tetanus, polio, and typhoid. In 1985, the Indian government added the measles vaccine under the Universal Immunization Programme (UIP) launch. The scale was established by combining the questionnaire proposed by the WHO in 2015 with a questionnaire from a related study [8,9]. The scale of the trust in the social environment displayed good reliability as reflected by a Cronbach’s α = 0.696.

A total of two subscales were extracted via factor analysis with principal component analysis, as reported in Table 2. Factors 1 and 2 were named trust in the social environment (Cronbach’s α = 0.887) and trust in vaccines (Cronbach’s α = 0.911), respectively, according to the factor loadings for the various question items after rotation. The KMO value of the factor analysis was 0.752, and the significance level *p*-value for the Bartlett’s test was 0.000. The two factors explained a total of 79.276% of the variance. There were reverse-coded questions in the question items (e.g., “Are you concerned that the COVID-19 vaccine may not be able to prevent COVID-19?”). Therefore, the scores for these questions were processed before calculation; that is, the response scores for the questions were 8 minus the original scores. After the questionnaires were completed, two values were obtained for trust in the social environment and trust in vaccines; the value for the former is the average score of the individual questions in the section of trust in the social environment and that for the latter is the average score of the individual questions in the trust in vaccines section.

### 2.5. Reliability Analysis

Reliability testing of the overall questionnaire, information reception scale, and trust scale yielded the following Cronbach’s alpha values, as presented in Table 3.

As can be seen from Table 3, the alpha value of the overall questionnaire is 0.779, which is greater than 0.7, indicating sufficient internal consistency. The overall alpha value of the information reception scale is 0.799, with Cronbach’s alpha values of 0.819 and 0.806 for official and unofficial sources, respectively. Both of the alpha values are greater than 0.8, indicating that the information reception scale has good reliability. The overall alpha value of the trust scale is 0.671, with Cronbach’s alpha values of 0.889 for trust in the social environment and 0.909 for trust in vaccines. Both are greater than 0.6, indicating that the trust scale has good reliability.

### 2.6. Control Variables

The control variables are mainly demographic variables. A total of four demographic variables were selected, namely sex (0 = woman; 1 = man), age (1 = 18 to 24; 2 = 25 to 29; 3 = 30 to 39; 4 = 40 to 49; 5 = 50 to 59), highest education level (1 = junior high school and below; 2 = senior high school or specialized middle school or technical secondary school or vocational high school; 3 = junior college or bachelor’s degree; 4 = master’s degree and above), and occupation (1 = school student; 2 = civil servant or workers in public institutions; 3 = workers in state-owned enterprises; 4 = employees of private and foreign companies; 5 = other).

### 2.7. Research Procedure

Participants were guided by a guideword to fill out demographic variables, the source reception scale, the trust scale, and the COVID-19 vaccination intention scale in order. Data were collected and analyzed using SPSS 24.0 software, and the parallel mediation effects analysis was conducted using the Hayes approach (2013) and PROCESS Macro by performing a bias-corrected bootstrap procedure.

## 3. Results

### 3.1. Data Statistics and Measurements

In the analysis of demographic variables, the distribution of age, sex, occupation, and education of the surveyed population were demonstrated, as reported in Table 4.

Among the information sources of the COVID-19 vaccine, the frequency of information reception from official sources was significantly higher than that from unofficial sources, with M = 5.54, SD = 1.37 and M = 4.08, SD = 1.49 for official and unofficial sources, respectively. Of the official sources, the frequency of receiving “information from the government” (M = 5.68, SD = 1.499) is greater than the frequency of receiving information from “experts and scholars in the field of medicine” (M = 5.39, SD = 1.511). Among the unofficial sources, “chatting and communicating with family and friends” scored the highest (M = 4.84, SD = 1.649), followed by “opinions from the Internet other than experts and scholars” (M = 4.51, SD = 1.848) and “opinions from netizens on the Internet” (M = 4.30, SD = 1.819), whereas the information from “overseas institutions” (M = 3.73, SD = 1.956) and “unknown sources” (M = 2.99, SD = 1.930) were less frequent.

In the statistics on people’s trust in all aspects involved in vaccines, the level of trust in the social environment was significantly higher and more concentrated than in vaccines (M = 5.67, SD = 1.129). The level of trust in government (M = 5.80, SD = 1.256) was slightly higher than in medical personnel (M = 5.53, SD = 1.199), and the level of trust in vaccines was relatively low and more dispersed (M = 4.38, SD = 1.739). People’s willingness to be vaccinated was generally high (M = 78.15, SD = 22.354). A total of 28.4% of respondents chose the maximum value of 99 (will definitely get vaccinated) and 58.7% scored above 80, indicating that people’s vaccine hesitancy is not currently serious.

### 3.2. Analysis of Correlation between Source of Information Received, Trust, and Vaccination Intention

In the variance analysis of demographic factors, one-way ANOVA was conducted for age, education, and occupation with vaccination intention for the COVID-19 vaccine. Independent-samples *t*-test was conducted for sex with vaccination intention, and the *p*-value significance was more than 0.05 in all cases, indicating that there was no difference in the mean for each group of demographic factors, and there was no difference in vaccination intention among people of different age groups, highest education level, occupation, and sex. These demographic factors were not influential factors in vaccination intention.

As reported in Table 5, which showcases the significant correlation between official and unofficial sources, the results of the correlation analysis between sources, trust, and vaccination intention indicated that both types of sources (official and unofficial sources) and both types of trust (trust in the social environment and in vaccines) are significantly and positively correlated with vaccination intention. Thus, H1a, H1b, H2a, and H2b were all supported. In terms of the relationship between sources and trust, both official and unofficial sources were significantly and positively related to trust in the social environment. However, in terms of trust in vaccines, official sources were not significantly related, whereas unofficial sources were significantly and negatively related to trust in vaccines. Thus, H3a, H3b, H3c, and H3d were not supported.

### 3.3. Information Reception from Different Sources and Vaccination Intention for COVID-19 Vaccine

The mediation effects analysis of trust examined whether the reception of information from different sources has a direct effect on vaccination intention and the mediating role of trust between the reception of information and vaccination intention. Bootstrap was used for the analysis, and the results are reported in Table 6.

The results of the regression analysis revealed that information receptions from official and unofficial sources were significant positive predictors of trust in the social environment (β = 0.343, *p* < 0.01; β = 0.169, *p* < 0.01), but they were not the significant predictors of trust in vaccines (β = −0.013, *p* > 0.01; β = −0.071, *p* > 0.01), as presented in Table 7 and Table 8. If the information reception from official sources, trust in the social environment, and trust in vaccines were combined to predict vaccination intention, all of the three factors had significant positive predictive effects on vaccination intention (β = 2.293, *p* < 0.01; β = 6.849, *p* < 0.01; β = 2.426, *p* < 0.01). If the information reception from unofficial sources, trust in the social environment, and trust in vaccines were combined to predict vaccination intention, all the three factors were significant positive predictors of vaccination intention (β = 1.441, *p* < 0.01; β = 7.611, *p* < 0.01; β *= 2*.424, *p* < 0.01).

As presented in Table 9 and Table 10, the mediating effects were further tested using the bias-corrected nonparametric percentile bootstrap method. The mediating effects were generated through two mediation chains: the indirect effect 1 (2.341) consisting of information reception from official sources → trust in the social environment → vaccination intention for the COVID-19 vaccinate, and indirect effect 3 (1.297) consisting of information reception from unofficial sources → trust in the social environment → vaccination intention for the COVID-19 vaccine. The bootstrap results on the 95% level of confidence for all confidence intervals did not contain zero, indicating that the mediating effect of trust in the social environment on the relationship between reception of information from official and unofficial sources and vaccination intention was significant. The bootstrap results on the 95% level of confidence for all confidence intervals for indirect effect 2 (−0.031) and indirect effect 4 (−0.171) contained zero, indicating that the mediating effect of trust in vaccines on the relationship between reception of information from official and unofficial sources and vaccination intention was not significant. Thus, H4 was partially supported, and trust in the social environment had a mediating effect on the relationship between information reception and vaccination intention. 

## 4. Discussion

**Figure 1:**
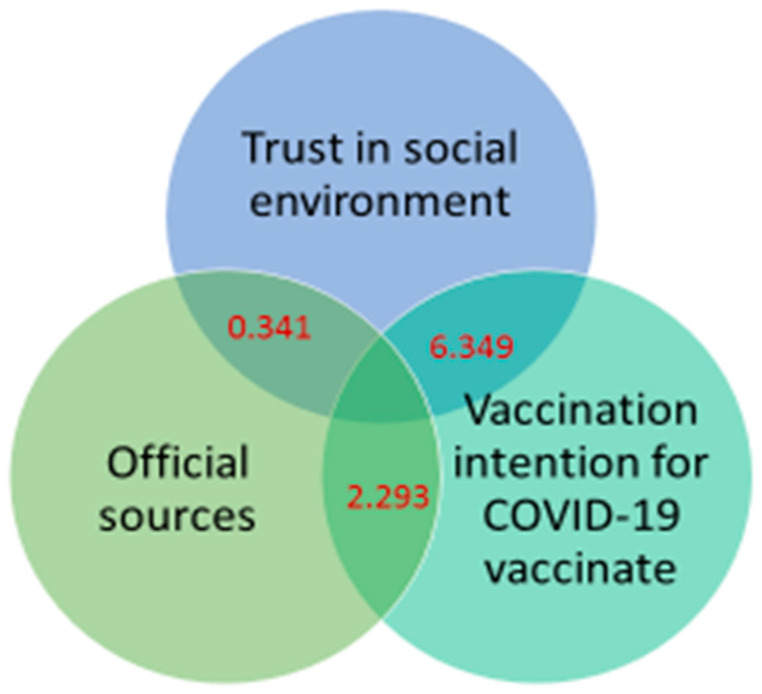
Schematic representation of mediating role of trust in social environment on the relationship between official sources of information reception and vaccination intention for COVID-19 vaccine.

Previous studies have revealed significant vaccine hesitancy in the United States and Canada [7]. In previous studies using the vaccine hesitancy model, researchers have typically focused on the trust in vaccines, such as the trust in their safety and efficacy [7,8,9]. The current study presented a different picture of vaccine hesitancy in India; people’s perceptions of the COVID-19 vaccine are favorable, and vaccine hesitancy has not been widespread. This confirms that trust in the social environment plays an important role in influencing people’s conduct in terms of health protection from the perspective of the prevention and control of COVID-19. Thus, exploring the strengths of India’s vaccination policies and information dissemination strategies may provide an opportunity to inform global vaccination efforts and contribute to global pandemic prevention and control. Previous studies have suggested that interpersonal communication and the dissemination of information on social media exacerbate people’s trust in false information [23]. In contrast, this study demonstrated that unofficial sources are also indispensable health information publishers and disseminators during the current pandemic, and they help people to increase social trust and thus proactively engage in scientific health protection. In previous studies, trust has always been a mediator of interest in the relationship between behavior and intention. In addition, research on the mediators of information reception and vaccination intention has been mainly limited to the trust in the vaccine itself (safety, efficacy) [28], with little consideration of trust in the social environment. Against the background of the special environment of India, this study demonstrated that trust in the environment is an important channel linking people’s information reception and vaccination intention and taps into a new pathway for health information dissemination (as presented in Figure 1).

Based on the above findings, this study expects to provide new ideas for health communication research. First, the perception of environment is an important basis for public health actions, which can significantly influence people’s health actions. The trust in the environment drives people to take active and effective health protection initiatives. This expands the theoretical research horizon of health communication and broadens the dimension of research on health communication paths.

Second, the source of information is an important aspect in influencing public health actions, and a new pattern of health information release and dissemination is gradually taking shape. Unofficial sources have become active promoters of people’s health protection initiatives, and a new pattern of multiple dissemination of health information is emerging. In India, scholars, experts, and government officials are now utilizing various channels and discourses to disclose information and popularize health information to the public, which has increased people’s willingness to take health protection measures. Therefore, the advantages of both official and unofficial publishers should be continuously brought into play to help health communication. Third, the development of multiparty trust is necessary in health programs promoted and participated in by the media. The release and promotion of health information should focus on persuasive methods and entry angles and improve the power and influence of information dissemination in terms of knowledge popularization, news reporting, and counter-rumor; and trust in all aspects of the environment should be built to carry out scientific and effective health protection.

The social determinants have been established to hinder the progress of vaccination efforts during the previous vaccination studies. In the United States, flu vaccinations are around 60% for children and lower among adults (45%). The role of risk perception has been identified in previous studies as in our study [29]. As seen in the literature, individuals who appraise that they are vulnerable to COVID-19 or who feel it as a life-threatening illness are more likely to get vaccinated in comparison with those who neglect or disagree COVID-19 to be life-threatening. 

Literature studies have leveraged the relationship between critical information socially provided and vaccination intentions and behavior [30]. The social trust is dependent on the information provided by the government, medical authorities, and health-care facilities. A study conducted in Italy outlined that COVID-19 vaccine acceptance is associated with trust or mistrust in biomedical research [31]. The role of political whirlpool is highly linked to every initiative or action that the government initiates in combatting COVID-19 within a community or society. Specific political principles used by individual political parties have shown to cause more rifts in vaccine hesitancy rates mainly due to the projection of false information by the conservative media [22]. Numerous studies and assumptions have frequently documented extensive anti-vaccine schemes and reports in the social media [20,21]. The defiance for public health messaging in such an environment is not simple given the “wild west” nature of social media despite recent attempts by social media platforms to flag anti-vaccine content [13,24]. One potential pathway is to increase trust in science and scientists and communicate the standards and regulatory process for vaccine approvals. Yet, a typical establishment approach based on science and facts is unlikely to have a direct influence given that anti-vaccine sentiments are strongly influenced by emotions, often when preferences are driven by “affect heuristic” [25]. It is critical to develop strategies to increase trust while countering the anti-vaccine influences drawing from strategic communication principles. A series of recent studies have documented the unequal impact of COVID-19-related impact on morbidity and mortality on “vulnerable” groups and communities including people of color, immigrants, and those in low-wage occupations and lower incomes. Almost without exceptions, almost all these studies showed that the social, economic, and health burden is being faced by such groups [26,27,28]. The fact that there are education- or schooling-based variations in the likelihood of obtaining vaccines warrants a more directed and strategic approach. We need to understand the reasons for reluctance among people with low schooling and how to address them. In addition, studies have documented inequalities in communication that deter access to processing of and the capacity to act on information among different social classes, which need to be addressed in the context of COVID-19 [19,30]. In what appears to be counterintuitive, people who are working or employed are more likely to be reluctant to obtain a vaccine compared with retired and student populations. While the issue needs to be explored further, the immediate public health implication is targeting this population in public health communications.

Limitation: The study was focused on determining sources, trust, and vaccination intention of COVID-19 vaccines. The study did not explore in detail the relation of geographic distribution, economic status, and literacy rates to vaccine hesitancy. Race and ethnicity were avoided as India has a very sparse multirace population. Since India is a large country with a high population, our study was very specific in confining to its aim to avoid statistical bias generated during multiple variables. 

## 5. Conclusions

A new path was found to influence the mechanism of vaccination intention. Specifically, trust in the social environment mediates the relationship between information reception and vaccination intention. This study demonstrated that trust in environment is an important channel linking people’s information reception and vaccination intention, explores a new path for health information communication, and attempts to provide new ideas for health information dissemination and promotion.

## Figures and Tables

**Table 1:** Factor analysis of information reception sources.

Measurement Question Item	Factor 1	Factor 2
Source: Internet users’ opinions in the network	0.853	
Source: unknown source	0.837	
Source: Internet opinion leaders other than experts and academics (e.g., Internet-verified celebrity from Chinese Weibo platform)	0.728	
Source: overseas institutions (e.g., foreign governments, overseas research institutions, overseas media)	0.667	
Source: chatting and communicating with family and friends	0.632	
Source: government access to information		0.949
Source: public voices of experts and scholars in medicine and other fields		0.879

Note: Extraction method: principal component analysis; rotation method: varimax with Kaiser normalization.

**Table 2:** Trust factor analysis.

Measurement Question Item	Factor 1	Factor 2
Do you trust the information provided by government departments about the COVID-19 vaccine?	0.891	
Do you trust the vaccine recommendations provided by your doctor?	0.869	
Do you believe that the government will make a decision in your best interests as to what kind of vaccine to provide?	0.861	
Can you feel that the doctor who serves you cares about what is best for your health?	0.839	
Are you worried that you may have serious side effects due to COVID-19 vaccination?		0.919
Are you concerned that the COVID-19 vaccine may not be safe to administer?		0.899
Are you concerned that the COVID-19 vaccine may not be able to prevent COVID-19?		0.881

Note: Extraction method: principal component analysis; rotation method: varimax with Kaiser normalization.

**Table 3:** Cronbach’s alpha values for each scale.

Cronbach’s Alpha Value	
Overall questions	0.779
Information reception scale	0.799
Trust scale	0.671

**Table 4:** Demographic variables.

Determinants	No. of Respondents	Percentage (%)
**Age**		
18 to 25 years	394	22.54
26 to 30 years	191	10.93
31 to 40 years	403	23.05
41 to 50 years	432	24.71
51 to 60 years	328	18.76
**Sex**		
Men	862	49.31
Women	886	50.69
**Occupation**		
School student	299	17.11
Government employee	301	17.22
Employee in state-owned companies	223	12.76
Employee in private and foreign companies	564	32.27
Other occupations	361	20.65
**Level of Education**		
Primary school	168	9.61
High school	434	24.83
Undergraduates	1056	60.41
Postgraduates and above	90	5.15

**Table 5:** Correlations between variables.

	Official Source	Unofficial Source	Trust in Social Environment	Trust in Vaccines	Vaccination Intention
Official source	1				
Unofficial source	0.391 *	1			
Trust in social environment	0.419	0225	1		
Trust in vaccines	−0.015	−0.068	0.121	1	
Vaccination intention	0.167	0.167	0.421	0.225	1

Note: * *p <* 0.01.

**Table 6:** Mediation effects testing model.

Pathways		
Model 1	Pathway 1	Official sources—trust in the social environment—vaccination intention
	Pathway 2	Official sources—trust in vaccines—vaccination intention
	Pathway 3	Unofficial sources—trust in the social environment—vaccination intention
Model 2	Pathway 4	Unofficial sources—trust in vaccines—vaccination intention

**Table 7:** Regression Model 1 for vaccination intention for COVID-19 vaccine.

Regression Equation (1)		Overall Fit Index		Significance of Regression Coefficients	
Target Variables	Predictor Variables	R	F	β	t
Trust in the social environment	Official sources	0.417	286.95	0.343	17.021 *
Trust in vaccines	Official sources	0.011	0.128	−0.013	−0.355
	Official sources			2.293	5.331 *
Vaccination intention	Trust in the social environment				
	Trust in vaccines	0.476		2.426	7.861 *

Note: * *p* < 0.01.

**Table 8:** Regression Model 2 for vaccination intention for COVID-19 vaccine.

Regression Equation (2)		Overall Fit Index		Significance of Regression Coefficients	
Target Variables	Predictor Variables	R	F	β	t
Trust in the social environment	Unofficial sources	0.221	68.759	0.169	8.289 *
Trust in vaccines	Unofficial sources	0.059	4.541	−0.071	−2.129
	Official sources			1.441	3.744 *
Vaccination intention	Trust in the social environment	0.467	126.221	7.611	15.379 *
	Trust in vaccines			2.424	7.781 *

Note: * *p* < 0.01.

**Table 9:** Results of bootstrapping analysis for mediating effects of trust factors.

Model 1	Effect Value	Boot Standard Error (SE)	Boot CI of the Lower Limit (LLCI)	Boot CI of the Upper Limit (ULCI)	Relative Effect Value
Indirect effect 1	2.341	0.297	1.799	2.939	50.82%
Indirect effect 2	−0.031	0.094	−0.215	0.156	
Direct effect	2.289	0.429	1.447	3.137	49.85%
Overall effect	4.498	0.427	3.759	5.429	100%

**Table 10:** Results bootstrapping analysis for mediating effects of trust factors.

Model 1	Effect Value	Boot Standard Error (SE)	Boot CI of the Lower Limit (LLCI)	Boot CI of the Upper Limit (ULCI)	Relative Effect Value
Indirect effect 3	1.297	0.211	0.899	1.715	50.52%
Indirect effect 4	−0.171	0.093	−0.353	0.011	
Direct effect	1.439	0.385	0.686	2.194	56.13%
Overall effect	2.561	0.417	1.751	3.379	100%

## Data Availability

Data and the copies of the questionnaire are available upon reasonable request to the corresponding author.

## Supplementary Materials

The following supporting information can be downloaded at: https://www.mdpi.com/article/10.3390/vaccines10101749/s1, File S1: Survey form.
