# Supplementary material for: Social Environmental Predictors of COVID-19 Vaccine Hesitancy in India: A Population-Based Survey
Source: Vaccines (Basel). 2022 Oct 19;10(10):1749. doi: 10.3390/vaccines10101749 (PMC9611416; doi:10.3390/vaccines10101749)
Supplement: Supplementary file 1 [file vaccines-10-01749-s001.zip › vaccines-1934366-supplementary.pdf]

Survey form:

Kindly fill/mark all the components of this survey form		
**1. Age**		
**2. Sex** Male<br>Female		
**3. Occupation**<br>School student<br>Government employee<br>Employee in state owned company<br>Employee in private and foreign companies<br>Other occupations		
**4. Level of Education**<br>Primary school (Standard 1-7)<br>High school<br>Undergraduate<br>Post graduate and above		
**5. Information reception about the COVID-19 vaccine:**<br><br>**Official source**<br>(1) information disclosure by the government<br>(2) public voices of experts and scholars in medical and other fields<br><br>**Unofficial source**<br>(1) online opinion leaders (e.g., internet verified celebrities from the social media platform)<br>(2) internet users' opinions<br>(3) gossip<br>(4) overseas institutions (e.g., foreign governments, research institutions, media)<br>(5) chatting with family and friends, both online (e.g., WeChat, phone call) and offline (face-to-face).	Answer each of the question in the following frequency *(circle or tick on the number that mostly satisfies your answer):*<br>1-never<br>2-very rarely<br>3- Rarely<br>4-occasionaly<br>5-often<br><br>1 2 3 4 5 6 7<br><br>1 2 3 4 5 6 7<br><br>1 2 3 4 5 6 7<br><br>1 2 3 4 5 6 7<br>1 2 3 4 5 6 7<br>1 2 3 4 5 6 7<br><br>1 2 3 4 5 6 7	

6. Do you trust the information provided by government departments about the COVID-19 vaccine?	Yes/No	
7. Do you trust the vaccine recommendations provided by your doctor?	Yes/No	
8. Do you believe that the government will make a decision in your best interests as to what kind of vaccine to provide?	Yes/No	
9. Can you feel that the doctor who serves you cares about what is best for your health?	Yes/No	
10. Are you worried that you may have serious side effects due to COVID-19 vaccination?	Yes/No	
11. Are you concerned that the COVID-19 vaccine may not be safe to administer?	Yes/No	
12. Are you concerned that the COVID-19 vaccine may not be able to prevent COVID-19?	Yes/No	
